# Risk of recurrence and conditional survival in complete responders treated with TKIs plus or less locoregional therapies for metastatic renal cell carcinoma

**DOI:** 10.18632/oncotarget.8302

**Published:** 2016-03-23

**Authors:** Daniele Santini, Matteo Santoni, Alessandro Conti, Giuseppe Procopio, Elena Verzoni, Luca Galli, Giuseppe di Lorenzo, Ugo De Giorgi, Delia De Lisi, Maurizio Nicodemo, Marco Maruzzo, Francesco Massari, Sebastiano Buti, Emanuela Altobelli, Elisa Biasco, Riccardo Ricotta, Camillo Porta, Bruno Vincenzi, Rocco Papalia, Paolo Marchetti, Luciano Burattini, Rossana Berardi, Giovanni Muto, Rodolfo Montironi, Stefano Cascinu, Giuseppe Tonini

**Affiliations:** ^1^ Department of Medical Oncology, Campus Bio-Medico University of Rome, Rome, Italy; ^2^ Clinica di Oncologia Medica, Università Politecnica delle Marche, AOU Ospedali Riuniti, Ancona, Italy; ^3^ Dipartimento di Scienze Cliniche Specialistiche ed Odontostomatologiche, Clinica di Urologia, AOU Ospedali Riuniti, Ancona, Italy; ^4^ Oncology Unit I, Fondazione IRCCS, Istituto Nazionale dei Tumori, Milan, Italy; ^5^ Division of Medical Oncology II, Azienda Ospedaliero-Universitaria Pisana, Istituto Toscano Tumori, Pisa, Italy; ^6^ Department of Clinical Medicine, Medical Oncology Unit, Federico II University, Naples, Italy; ^7^ Istituto Scientifico Romagnolo per lo Studio e la Cura dei Tumori (IRST) - IRCCS, Meldola, Italy; ^8^ Sacro Cuore - Don Calabria Hospital, Negrar, Italy; ^9^ Medical Oncology I, Istituto Oncologico Veneto IOV, IRCCS, Padova, Italy; ^10^ Medical Oncology, Azienda Ospedaliera Universitaria Integrata, University of Verona, Verona, Italy; ^11^ Oncology Unit, University Hospital of Parma, Parma, Italy; ^12^ Department of Urology, Campus Bio-Medico University of Rome, Rome, Italy; ^13^ Niguarda Cancer Center, Ospedale Niguarda Ca' Granda, Milan, Italy; ^14^ IRCCS San Matteo University Hospital Foundation, Pavia, Italy; ^15^ Medical Oncology Unit Policlinico Sant'Andrea, Rome, Italy; ^16^ Section of Pathological Anatomy, Polytechnic University of the Marche Region, School of Medicine, AOU Ospedali Riuniti, Ancona, Italy

**Keywords:** renal cell carcinoma, complete responder patients, tirosin kinase inhibitor, risk of recurrence, conditional survival

## Abstract

**PURPOSE:**

We retrospectively analyzed the risk of recurrence and conditional Disease-Free Survival (cDFS) in 63 patients with complete remission during treatment with tirosin kinase inhibitor (TKI), alone or with local treatment in metastatic renal cell carcinoma.

**RESULTS:**

37% patients achieve CR with TKI alone, while 63% with additional loco-regional treatments. 49% patients recurred after CR, with a median Disease free survival of 28.2 months. Patients treated with multimodal approaches present lower rate of recurrence (40% vs 61%) and longer Disease free survival compared to patient treated with TKI alone (16.5 vs 41.9 months, *p*=0.039).Furthermore the rate of recurrence was higher in patients with brain (88%), pancreatic (71%) and bone metastasis (50%). Patients who continued TKI therapy after complete response had a longer disease free survival than patients who stopped therapy, although the difference was not significant (42.1 vs 25.1 months, *p*=0.254). 2y-cDFS was better in patients treated with multimodal treatment and who continued TKIs than the other patient arms.

**CONCLUSIONS:**

The prognostic value of CR depends on the site where was obtained and how was obtained (with or without locoregional treatment). Cessation of TKI should be carefully considered in complete responder patients.

## INTRODUCTION

Renal cell carcinoma (RCC) is the most frequent type of kidney neoplasm in adults. RCC presents as metastatic a diagnosis in almost 30% of patients and approximately 20% metastasizes after radical or partial nephrectomy [1] [2]. Although the prognosis of RCC patients has been dramatically increased by the introduction of anti-vascular endothelial growth factor receptor (VEGFR) tyrosine kinase inhibitors (TKIs), anti-VEGF antibodies and mTOR inhibitors, the rate of patients who achieve complete remissions (CRs) is still poor [3]. The integration of these therapies with locoregional approaches is often proposed as a valid therapeutic option and should be evaluated case-by-case in order to optimize the outcome of RCC patients.

In 2012, Dr Albiges and her group described the results of a multicenter retrospective analysis including 64 patients who achieved CR during treatment with sunitinib or sorafenib administered alone or with local approaches. In this study, 53 patients (83%) stopped treatment after CR; of them, 29 patients (17 who had obtained CR with TKIs alone and 12 with additional local treatments) were still in CR at time of analysis [4].

Based on these results, several open questions still remain on: how can we early recognize patients who will achieve CR with targeted agents? What factors associate with the risk of recurrence after CR? Should we continue treating patients with targeted agents after CR? The answers to these questions will direct personalized strategies for mRCC patients and reduce the risk of recurrence after CR.

This multicenter retrospective analysis investigated prognostic factors and risk of recurrence in mRCC patients in CR after first-line targeted agents alone or in association with locoregional therapy. Secondary goal was to evaluate the risk of recurrence and conditional survival related to drug interruption vs continuation after CR.

## RESULTS

### Overall study population

Sixty three patients with CRs were enrolled in this analysis. The median follow-up time was 20.2 months (IQR 8.5-33.5). Forty-six of them were males (73%). Median age was 58 years (range 49–65 years). Tumor histology was predominantly clear cell (87%); 25 patients (40%) were metastatic at diagnosis. Lung (43%) and mediastinal and/or abdominal lymph nodes (30%) were the most frequent metastatic sites; 30 patients (48%) had more than one site of metastasis. Prognostic categories using MSKCC criteria were good in 40 pts (63%), intermediate in 23 patients (37%), whilst no patients had poor risk features. One hundred and seventy-nine patients were used as a control group. Complete patient demographics are shown in Table 1.

**Table 1:** Clinico-pathological characteristics of the overall population

N (%)	CR 63 patients	Non-CR 179 patients	*p-value*
**Gender**			0.265
Male	46 (73)	117 (65)	
Female	17 (27)	62 (35)	
**Median age (IQR)**	58 (49-65)	66 (57-87)	**<0.001**
**Karnofsky Performance Status at I line**			**<0.001**
≥90	56 (89)	30 (17)	
<90	7 (11)	149 (83)	
**T Stage**			0.073
T1	15 (24)	25 (14)	
T2	12 (19)	35 (19)	
T3	32 (51)	87 (49)	
T4	4 (6)	32 (18)	
**Fuhrman grade**			0.067
G1	9 (14)	25(14)	
G2	27 (43)	46 (25)	
G3	21(33)	87 (49)	
G4	6 (10)	21 (12)	
**Metastasis at RCC diagnosis**			0.810
M0	38 (60)	111 (62)	
M1	25 (40)	68 (38)	
**Histology**			0.643
Clear cell RCC	55 (87)	152 (85)	
Non-clear cell RCC	8 (13)	27 (15)	
**Sarcomatoid aspects**			0.080
No	60 (95)	151 (84)	
Yes	3 (5)	28 (16)	
**Previous nephrectomy**			**0.002**
Yes	55 (87)	120 (67)	
No	8 (13)	59 (33)	
**Metastatic sites at start of first line therapy**			**0.002**
Bone	9 (14)	66 (37)	**< 0.001**
Lymph-nodes	19 (30)	91 (51)	**0.004**
Lung	27 (43)	122 (68)	**<0.001**
Liver	8 (13)	51 (28)	**0.012**
Pancreas	7 (11)	7 (4)	0.035
Adrenal glands	9 (14)	19 (11)	0.433
Contralateral kidney	10 (16)	1 (1)	**<0.001**
Brain	8 (13)	24 (13)	0.886
**N metastatic sites**			**<0.001**
1	33 (52)	42 (24)	
>=2	30 (48)	137 (76)	
**MSKCC**			
Good risk	40 (63)	27 (15)	**<0.001**
Intermediate risk	23 (37)	140 (78)	**0.050**
Poor risk	0	12 (7)	—

CR = Complete remission; IFN-α = Interferon-α; MSKCC = Memorial Sloan Kettering Cancer Center; RCC= renal cell carcinoma

Comparing CR vs non-CR patients, age (Kruskal-Wallis *p*<0.001) and Karnofsky Performance Status (Kruskal-Wallis *p*<0.001) resulted significantly different between the two groups. No differences were found according to gender (*p*=0.260), tumor histology (*p*=0.643) and sarcomatoid differentiation (*p*=0.08). The absolute number of metastatic sites at start of first-line therapy was significantly higher in non-CR patients (*p*<0.001), as well as the number of patients with good (*p*<0.001) or intermediate (*p*=0.050) MSKCC criteria.

Finally, a significant difference in the proportion of metastatic sites were observed for bone, mediastinal and/or abdominal lymph nodes, lung and liver, which were more present in non-CR patients, whilst metastases to contralateral kidney were more frequent in patients with CR (Table 1).

### CR patients: locoregional approaches, drug interruptions and risk of recurrence

Fifty-one patients (81%) were treated with sunitinib, 9 with pazopanib (14%), 3 with bevacizumab and IFN-α (5%). Among them, dose reductions were performed in 28 patients (44%).

In 23 patients (37%), CR was obtained by TKI therapy alone, while 40 patients (63%) underwent additional loco-regional treatments, including surgery (88%), radiotherapy (8%) or both (4%). CR of brain metastases was obtained by radiotherapy and TKI therapy. After achieving CR, first-line TKI therapy was interrupted in 51 patients (81%) and continued in 12 patients (19%). Thirty-one patients (49%) recurred after CR. Of them, 27 had interrupted TKI therapy at CR (27/51, 53%) and 4 continued treatment after achieving CR (4/12, 33%). At recurrence, 15 of the 27 patients who had stopped TKI therapy was re-treated with the same TKI (56%), while the other 12 patients were treated with the another TKI (26%) or everolimus (5%). Four of the 15 patients who restarted therapy with the same agent do progressed during treatment, while only 1 patient progressed among the 12 patients who changed therapy. Complete detailed CR and recurrence characteristics are presented in Table 2.

**Table 2:** Locoregional approaches, drug interruptions and risk of recurrence

	N (%)
**First-line therapy**	
Pazopanib	9 (15)
Sunitinib	51 (81)
Bevacizumab + IFN-α	3 (3)
**Response to first-line TKI**	
CR without additional treatment	23 (37)
Additional loco-regional treatment	40 (63)
**Type of locoregional treatment**	
Metastasectomy	35 (88)
Radiotherapy	3 (8)
Combination therapy (surgery + radiotherapy)	2 (4)
**Recurrence after CR**	
Yes	31 (49)
No	32 (51)
**Suspension of TKI after CR**	
Yes	51 (81)
No	12 (19)
**Therapy at recurrence after CR**	
Same TKI used as first-line	15 (56)
Different TKI	7 (26)
Everolimus	5 (19)

**Figure 1:**
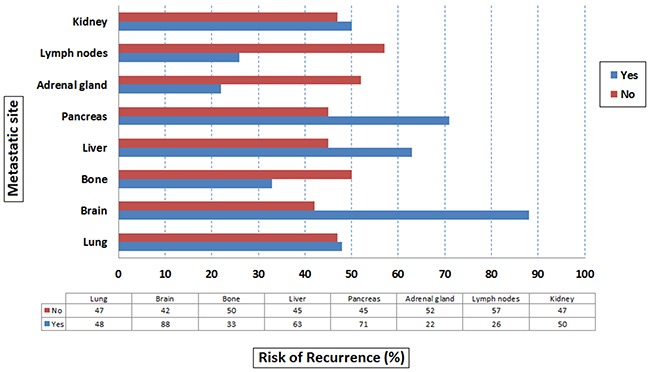
Risk of recurrence by metastatic site

Among the 31 patients who recurred after CR, 14 (45%) relapsed in the same metastatic sites in which they had achieved CR, whilst 17 (55%) recurred in different sites. Furthermore, we analyzed the recurrence rate according to the site of metastases at the start of first- line therapy. We showed that the rate of recurrence was higher in patients with brain (88%), pancreatic (71%) and liver metastasis (63%) (Figure 1). In addition, the rate of recurrence was lower in patients with only one site of metastasis compared to ≥ 2 sites (41% vs 52%), while was comparable in good risk vs intermediate risk patients (48% vs 48%). Notably, the rate of recurrence was lower in patients who achieved CR with TKIs and locoregional interventions compared to patients treated with only TKIs (40% vs 61%).

### Outcome analyses: disease-free survival and overall survival

**Figure 2:**
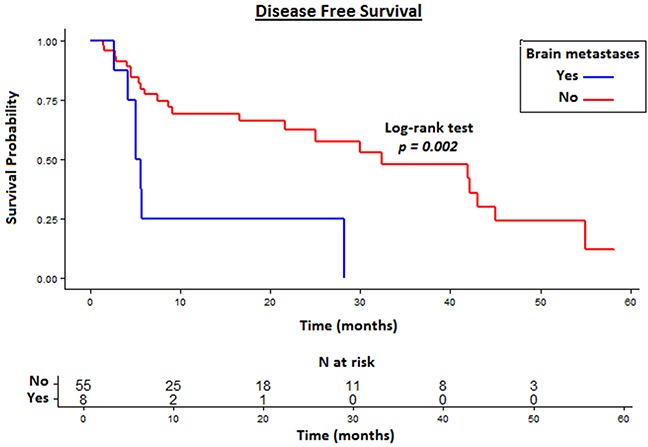
Disease Free Survival (DFS) in patients with CR of brain metastases compared to other metastatic sites

In patients with CR, median DFS was 28.2 months (95% CI 16.5–43.1). We further analyzed the median DFS based on the presence or absence of different metastatic sites. Patients with CR of brain metastases had significant shorter DFS compared to patients with CR of other metastatic sites (5.3 vs 32.4 months, *p*=0.002) (Figure 2). Otherwise, a significant difference was not found for lung (32.4 vs 28.2 months, *p*=0.534), liver (7.3 vs 32.4 months, *p*=0.073), adrenal gland (42.1 vs 25.1 months, *p*=0.184), bone (42.1 vs 25.1 months, *p*=0.093), pancreatic (5.6 vs 29.2 months, *p*=0.058), mediastinal and/or abdominal lymph nodes (25.1 vs 28.2 months, *p*=0.304) and contralateral kidney (28.2 vs 29.9 months, *p*=0.809). By stratifying patients for the number of metastatic sites (1 vs ≥2) the median DFS was 32.4 vs 21.6 months (*p*=0.288). Similarly, no difference was found in terms of DFS between patients with good vs intermediate risk criteria (28.2 vs 29.9 months, *p*=0.879).

**Figure 3:**
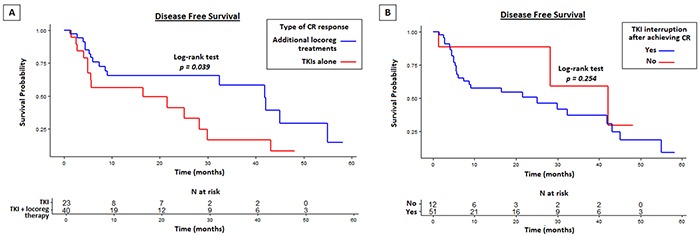
Disease free survival (DFS) in CR patients with or without locoregional approaches to TKI therapy **A.** and on TKI interruption/continuation after achieving CR **B.**

Patients who achieved CR with the addition of loco-regional interventions had a longer DFS compared to patients who received TKI alone (41.9 vs 16.5 months, *p*=0.039) (Figure 3A). Interestingly, patients who continued TKI therapy after CR had a longer DFS than those who interrupted TKI administration. The difference was not statistically significant due to the different number of patients in the two groups (42.1 vs 25.1 months, *p*=0.254) (Figure 3B). Patients who experienced dose reductions during first-line therapy had comparable DFS compared to patients who maintained the standard doses (32.4 vs 25.1 months, *p*=0.855).

Median OS from the start of first-line therapy and from CR were not reached due to the fact that only 4 patients (6%) were dead at time of this analysis.

### Univariate and multivariate analyses for DFS

At univariate analysis, the presence of metastases to pancreas (*p*=0.058; HR=2.54, 95% CI: 0.94−6.88), brain (*p*=0.002; HR=3.74, 95% CI: 1.52−9.21) and liver (*p*=0.073; HR=2.41, 95% CI: 0.89−6.48) and high Fuhrman grade (*p*=0.027; HR=2.12, 95% CI 1.37-3.15) were significantly associated with poor DFS. The addition of locoregional treatment to achieve CR (*p*=0.048; HR=0.47, 95% CI: 0.23−0.98) and the presence of metastases to adrenal gland (*p*=0.183; HR=0.39, 95% CI: 0.09−1.65) or bone (*p*=0.106; HR=0.37, 95% CI: 0.11−1.24) were correlated with significantly longer DFS. None of the other clinical variables, such as age, T stage, MSKCC group, number of metastatic sites and TKI interruption at CR were significantly associated with the outcome of CR patients. At multivariate analysis, the presence of liver (*p*=0.002; HR=4.13, 95% CI: 1.65−10.34) or brain metastases (*p*=0.045; HR=2.81, 95% CI: 1.02−7.69) and high Furhman grade (*p*=0.040; HR=1.98, 95% CI 1.36-5.65) were independent predictors of worst DFS (Table 3).

**Table 3:** Univariate and Multivariate analyses of predictors of DFS in patients with CR

Disease Free Survival	Univariate Cox regression		Multivariate Cox regression	
	HR (95% CI)	*p-value*	HR (95% CI)	*p-value*
**Age**				
(<60 y vs>60 y)	1.61 (0.77-3.34)	0.206		
**Fuhrman grading**	2.12 (1.37-3.15)	**0.027**	1.98 (1.36-5.65)	**0.040**
**T stage at diagnosis**	0.61 (0.29-1.27)	0.187	0.91 (0.40-2.91)	0.803
**Metastatic at diagnosis**				
No vs Yes	1.18 (0.56-2.48)	0.662		
**N. of metastatic sites**				
1 site vs >1 site	1.49 (0.71-3.10)	0.289		
**MSKCC risk group**				
Low risk vs Intermediate risk	1.06 (0.50-2.24)	0.885		
**Type of CR**				
Surgery+TKIs vs TKIs alone	0.47 (0.23-0.98)	**0.048**	0.80 (0.34-1.87)	0.603
**TKI interruption at CR**				
Yes vs No	1.25 (0.54-2.94)	0.602		
**Metastatic sites**				
Lung	0.79 (0.38-1.65)	0.534		
Pancreas	2.54 (0.94-6.88)	**0.058**	1.55 (0.43-5.53)	0.499
Adrenal gland	0.39 (0.09-1.65)	**0.183**	0.50 (0.11-2.27)	0.371
Bone	0.37 (0.11-1.24)	**0.106**	0.46 (0.14-1.58)	0.220
Brain	3.74 (1.52-9.21)	**0.002**	4.13 (1.65-10.34)	**0.002**
Liver	2.41 (0.89-6.48)	**0.073**	2.81 (1.02-7.69)	**0.045**
Lymph nodes	0.60 (0.23-1.59)	0.308		
Controlateral kidney	1.13 (0.42-2.99)	0.813		

Significant values are reported in bold. DFS= disease free survival, CR= complete remission

Univariate and multivariate analyses were not performed for OS due to the small number of events.

### Conditional disease-free survival and overall survival

**Figure 4:**
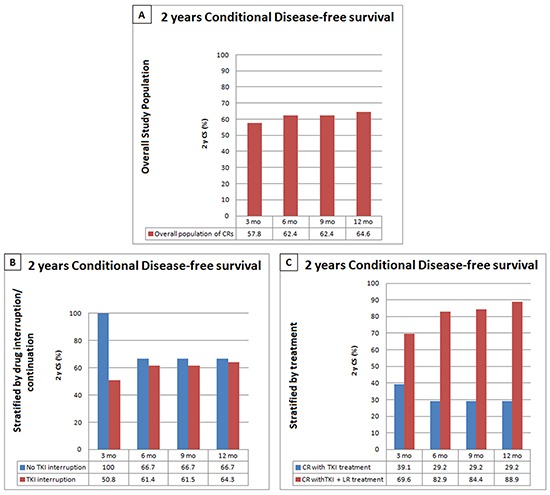
Conditional Disease Free Survival (cDFS) in the overall CR population **A.** and stratified by drug interruption/continuation after CR. **B.** or treatment received (TKI versus TKI plus locoregional treatment) **C.**

For the overall population of complete responders, DFS at 2 years was 56.5%. Considering the increasing timepoints investigated, the survival advantage was +1.3 (2%) at 3 months, +5.9 (10%) at 6 and 9 months, +8.1 (14%) at 12 months. Stratifying by treatment received, 2y survival probability was 41.1% in patients treated with TKIs alone and 65.7% for patients treated with TKIs plus locoregional treatments. In the first group of patients, no survival advantages were seen after time, as shown by the data on cDFS presented in Figure 4, while for patients undergoing associated locoregional treatments a gain in survival rates of 3.9 (6%), 17.2 (26%), 18.7 (28%) and 23.2% (35%), respectively at 3, 6, 9, 12 months survival time was observed.

The effect of treatment suspension was also investigated under a cDFS fashion. Even though a 2 years cDFS reduction with time was observed, treatment prosecution, compared to suspension, was associated to a better cDFS at all investigated timepoints.

## DISCUSSION

In this study, we first investigated the factors associated with the risk of recurrence in RCC patients with CR developed during first line therapy. We showed that the risk of recurrence after CR varies among the different metastatic sites that develop CR. Indeed, the presence of brain, bone or pancreatic metastases, even if obtained by TKIs alone or with local treatments, was associated with the highest risk of recurrence in patients with CR. In addition, the presence of brain or liver metastases was a predictor of worst DFS at multivariate analysis. The chance of CR seems to be influenced more by the site of metastasis than by the number of metastatic sites. In addition, our data showed that TKI interruption at CR was not a predictor of DFS at univariate and multivariate analysis, in accordance with the recent results from the registry-based analysis led by Buchler et al [11]. Taken together, these data support a personalized follow-up for mRCC patients based on the metastatic sites in CR.

In our study, 51 patients stopped therapy once in CR. Differently from the study led by Albiges et al. [4], the rate of patients who were still in CR after stopping therapy was lower in our study (47% vs 61%). This may be partially explained by the differences between the two study populations (i.e. not all of our CR patients underwent previous nephrectomy and no poor risk patients were included in our analysis).

In our study, patients that continued treatment after CR recorded 17 months of additional DFS. The small number of patients in both studies does not allow to definitively assess whether drug interruption can be safe and effective. Nevertheless our findings should be taken into consideration when dealing with CR mRCC patients. A significant advantage in terms of DFS was observed in patients treated with a multimodal approach compared to patients treated with TKI alone. Considering the low rate of recurrence and longer disease free interval in the group of patient treated with TKI and local therapy, multimodal treatment could be a valid approach to overcome tumor heterogeneity usually involved in TKI resistance.

Interestingly, dose reductions during TKI therapy were reported in 44% of CR patients and did not correlate with a higher rate of recurrence after CR. These findings should be considered to evaluate the choice of more tolerable strategies (i.e. reduced doses, alternative schedules) in patients in CR with TKIs. In addition, the potential of emerging immunotherapies, such as Immunocheckpoints inhibitors, as a maintenance therapy after achieving CR should be investigated in these patients. Indeed, Immunocheckpoints inhibitors can induce T cell–mediated memory response that is unattainable with TKI therapy, thus suggesting that the combination of these two different approaches may potentially increase the rate of patients without disease recurrence after obtaining CR with TKI therapy.

The early identification of patients who will develop CR or rapidly progress during first-line therapy represents a challenging issue in mRCC management. Clinicopathological data provide essential information in guiding treatment decisions, even if validated predictive biological factors associated with the response to targeted agents are still lacking. When comparing non-CR patients, we found that patients with CR were younger, presented a better Karnofsky Performance Status and a different metastatic distribution at the start of first-line therapy. These data suggest the employment of local approaches to achieve CR and emphasize the necessity for a multidisciplinary management of these patients.

However, there are several limitations to this study. First, its retrospective design, which may incur bias in data selection and analysis. Second, the lack of a central radiologist review of the CRs, which were separately evaluated and confirmed by the oncologists and the radiologists in each center. Furthermore, data on patient quality of life (QoL) would be an important integration for the results of our analysis. In addition, the study population was quite heterogeneous due to the presence of patients who had obtained a CR with or without locoregional therapies.

Despite these limitations, the present study shows that CR can be obtained by TKI therapy, either alone or in combination with local approaches. Drug continuation after CR should be carefully considered in RCC patients, even if perspective studies are dramatically needed to identify and validate predictive factors associated with the risk of recurrence after CR in this population.

## PATIENTS AND METHODS

### Study population

This was a retrospective, observational multicenter study including patients consecutively treated for metastatic RCC from March 2006 to May 2014 (i.e. not on clinical trials or experimental protocols) and presenting with CR (absence of clinically and/or radiologically identifiable signs of the disease) on first-line therapy, administered alone or in combination with local treatments (i.e. metastasectomy, radiotherapy, radiofrequency ablation and stereotaxis) in accordance with their treating physician's practice. Baseline characteristics were obtained from patients' clinical charts at admission from 13 Italian centers. Patients were collected consecutively to avoid selection bias and were excluded from the analysis if they had missing information about first-line therapy, sites of metastasis at the start of therapy, locoregional therapies, time to CR and recurrence. Patient follow-up was updated to May 2015.

Radiological CR was defined either with total body contrast-enhanced CT and/or MRI. Memorial Sloan Kettering Cancer Center (MSKCC) prognostic criteria [5], number and location of metastatic sites, type of CR (obtained by targeted therapy alone or by the addition of loco-regional approaches), drug interruption after complete response were considered as potentially relevant variables for differentiating the groups and for the purposes of outcome prediction.

CR patients were matched with a group of consecutive patients with non-CRs (partial responses, stable disease or progressive disease) to first-line therapy for metastatic RCC treated in the same period at the Campus Biomedico (Rome) and the AOU Ospedali Riuniti (Ancona).

### Statistical analysis

Overall Survival (OS) was defined as the time from CR after first-line treatment to death, irrespective of cause. Disease-Free Survival (DFS) was defined as the time from CR to disease recurrence. OS and DFS were evaluated via the Kaplan-Meier method and Mantel-Haenszel log-rank test was employed to compare survival among groups. We considered distribution of clinical variables within each of the two groups of patients (CR vs non-CRs). Homogeneity of the two groups relating to variables distributions was tested by means of chi-squared test for difference in proportions.

Variable tendency measures were reported as mean (95% CI) or median (IQR) according to their distribution. Uniform distribution of categorical variables between the two groups compared was evaluated by means of chi-squared test. Levels of continuous variables between the two groups were compared by means of Kruskal-Wallis test.

Median OS and DFS were estimated by means of Kaplan-Meier product-limit method, while the Mantel-Haenszel log-rank test was used for statistical inference in the formal comparison between groups. A Cox-regression model was applied to the data with a univariate and multivariate approach. The assumption of proportionality of hazards was assessed using the Therneau and Grambsch test of the Schönefeld residuals [6]. Variables not fitting a univariate regression analysis were excluded for the multivariate model. No-multicollinearity of the grouped co-variates was also checked. Significance level in the univariate model for inclusion in the multivariate final model was more liberally set at a 0.2 level, according to Hosmer *et al*. [7]. All other significance levels were set at a 0.05 value.

Conditional DFS (cDFS) is the conditional probability for a patient to survive without experiencing recurrence for an additional number of years. Kaplan-Meier lifetables were employed to estimate cumulative survival at six different timepoints (3, 6, 9, 12 months from diagnosis of CR) and further at 24 months + each of the timepoints. Two-years cDFS relative to each timepoint was then obtained by dividing the survival rate at each of the 24 + timepoints months by the survival rate at the baseline timepoints. In other words, cDFS (2y|timepoint)=S(timepoint+24)/S(timepoint) according to previous studies [8] [9] [10]. Comparison of changes in cDFS was further performed after stratification according to the most clinically relevant variables at univariate analysis.

All calculations were conducted by means the R Software v. 3.1.2 (The R Company- Vienna- Austria).

## References

[1] Athar U (2008). Gentile TC: Treatment options for metastatic renal cell carcinoma: a review. Can J Urol.

[2] Motzer RJ, Bander NH, Nanus DM (1996). Renal-cell carcinoma. N Engl J Med.

[3] Iacovelli R, Alesini D, Palazzo A, Trenta P, Santoni M, De Marchis L, Cascinu S, Naso G, Cortesi E (2014). Targeted therapies and complete responses in first line treatment of metastatic renal cell carcinoma. A meta-analysis of published trials. Cancer Treat Rev.

[4] Albiges L, Oudard S, Negrier S, Caty A, Gravis G, Joly F, Duclos B, Geoffrois L, Rolland F, Guillot A, Laguerre B, Legouffe E, Kohser F (2012). Complete remission with tyrosine kinase inhibitors in renal cell carcinoma. J Clin Oncol.

[5] Motzer RJ, Mazumdar M, Bacik J, Berg W, Amsterdam A, Ferrara J (1999). Survival and prognostic stratification of 670 patients with advanced renal cell carcinoma. J Clin Oncol.

[6] Grambsch P, Therneau TM (1994). Proportional hazards tests and diagnostics based on weighted residuals. Biometrika.

[7] Hosmer DW, Lemeshow S (1999). Interpretation of a fitted proportional Hazards Regression Model. Applied Survival Analysis: Regression Modeling of Time to Event Data.

[8] Skuladottir H, Olsen JH (2003). Conditional survival of patients with the four major histologic subgroups of lung cancer in Denmark. J Clin Oncol.

[9] Ploussard G, Shariat SF, Dragomir A, Kluth L, Xylinas E, Masson-Lecomte A, Rieken M, Rink M, Matsumoto K, Kikuchi E, Klatte T, Boorjian SA, Lotan Y (2014). Conditional survival after radical cystectomy for bladder cancer: evidence for a patient changing risk profile over time. Eur Urol.

[10] Ploussard G, Xylinas E, Lotan Y, Novara G, Margulis V, Rouprêt M, Matsumoto K, Karakiewicz PI, Montorsi F, Remzi M, Seitz C, Scherr DS, Kapoor A, Fairey AS, Rendon R (2015). Conditional survival after radical nephroureterectomy for upper tract carcinoma. Eur Urol.

[11] Buchler T, Bortlicek Z, Poprach A, Pavlik T, Veskrnova V, Honzirkova M, Zemanova M, Fiala O, Kubackova K, Slaby O, Svoboda M, Vyzula R, Dusek L (2015). Outcomes for Patients with Metastatic Renal Cell Carcinoma Achieving a Complete Response on Targeted Therapy: A Registry-based Analysis. Eur Urol.

